# Low-Osmolar vs. Iso-Osmolar Contrast Media on the Risk of Contrast-Induced Acute Kidney Injury: A Propensity Score Matched Study

**DOI:** 10.3389/fmed.2022.862023

**Published:** 2022-04-29

**Authors:** Taeho Lee, Won Ki Kim, Ae Jin Kim, Han Ro, Jae Hyun Chang, Hyun Hee Lee, Wookyung Chung, Ji Yong Jung

**Affiliations:** ^1^Division of Nephrology, Department of Internal Medicine, Gachon University Gil Medical Center, Incheon, South Korea; ^2^Division of Nephrology, Department of Internal Medicine, College of Medicine, Gachon University, Incheon, South Korea

**Keywords:** contrast media (CM), osmolality, acute kidney injury (AKI), coronary artery disease, contrast-induced acute kidney injury (CI-AKI), propensity score matching

## Abstract

**Objective:**

Among the various risk factors associated with contrast-induced acute kidney injury (CI-AKI), the importance of osmolality and viscosity is emerging among the characteristics of contrast media (CM) itself. High osmolality CM (HOCM) is deprecated and low osmotic pressure (LOCM) and iso-osmotic pressure (IOCM) are mainly used in clinical situations where the results of studies on their effect on the development of CI-AKI are contradictory. We evaluated the association between the type of CM and the risk of CI-AKI.

**Materials and Methods:**

A retrospective observational cohort study to analyze the effect of the type of CM on the development of CI-AKI. Using propensity score (PS) matching, 2,263 LOCM and IOCM groups were paired for analysis from 5,267 patients and fulfilled the inclusion criteria among 12,742 patients who underwent CAG between 1 January 2007, and 31 December 2016. LOCM included iopromide and iopamidol, IOCM was iodixanol. CI-AKI, which was the primary endpoint, was defined based on the Kidney Disease Improving Global Outcomes criteria within 48 h after exposure to the CM. A multivariable logistic regression analysis was used in the unmatched and matched cohorts, respectively. In addition, a stratified model on clinically important variables, including a high Mehran score (≥ 6), was also used in the matched cohort.

**Results:**

LOCM users showed an increased incidence of CI-AKI (11.7% vs. 9.3%; *p* = 0.006), but it lost statistical significance after PS matching (9.9% vs. 9.5%, *p* = 0.725). In multivariable analyses, the adjusted odds ratio for CI-AKI in the LOCM group were 1.059 [95% confidence interval (CI) = 0.875–1.282; *p* = 0.555] in unmatched cohort and 0.987 (95% CI = 0.803–1.214; *p* = 0.901) in matched cohort. These results were also consistent with the high-risk (high Mehran score) group.

**Conclusions:**

Although the role of CM types in the development of CI-AKI has been debated, our observation shows that the selection between LOCM and IOCM during CAG has no influence on the incidence of CI-AKI.

## Introduction

Contrast-induced acute kidney injury (CI-AKI) is an acute impairment of renal function after administration of intravascular iodine contrast media (CM) without other causes. CI-AKI is the third most common cause of in-hospital AKI (1), and has been related to an increase in mortality, long-term loss of kidney function, and need for renal replacement therapy (2, 3).

The risk of CI-AKI is affected by the patient- and procedure-related factors and the most important factor of CI-AKI is pre-existing renal impairment (4, 5). Among procedure-related factors, one of the modifiable risk factors is the characteristic of CM. Although the pathophysiology of CI-AKI has not been fully understood, many studies suggested that the characteristics of CM may play an important role in the occurrence of CI-AKI (4, 6). In particular, in terms of osmolality, previous studies have shown that high-osmolar CM (HOCM) is associated with an increased risk of CI-AKI than low-osmolar CM (LOCM) (7). Since then, iso-osmolar CM (IOCM), which is characterized by iso-tonicity with human plasma, had developed. Because initial studies showed that IOCM had less nephrotoxicity than LOCM (8, 9), the use of IOCM was expected to reduce the incidence of CI-AKI. However, these were small-sized studies and follow-up studies showed different results. There is no consensus on whether LOCM has more nephrotoxicity than IOCM as well as no large-scale study about this. Therefore, we analyzed the difference between LOCM and IOCM in the development of CI-AKI among patients who underwent diagnostic or interventional coronary catheterization procedures.

## Materials and Methods

### Study Design and Participants

**Figure 1:**
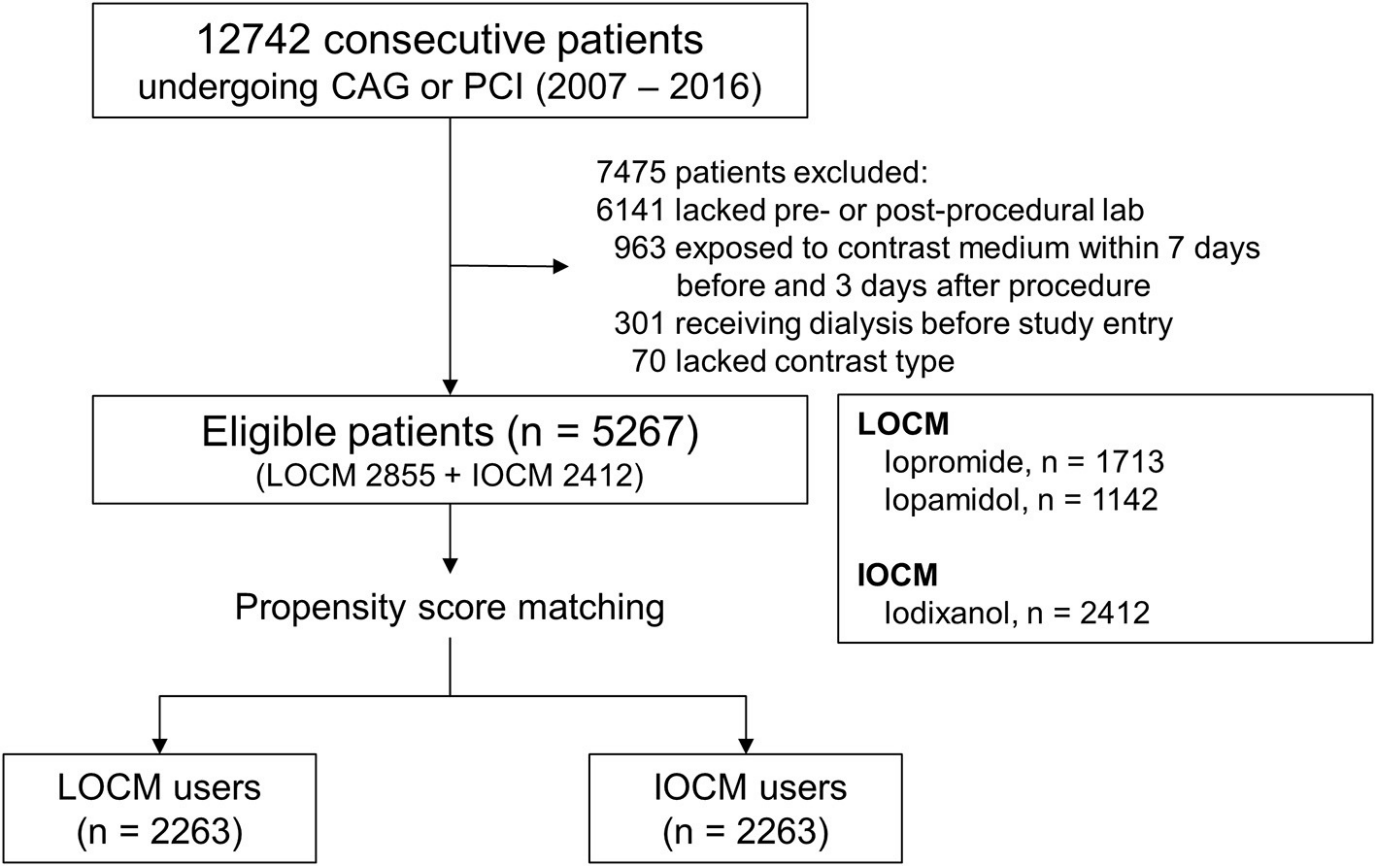
Cohort formation. CAG, coronary angiography; PCI, percutaneous coronary intervention; LOCM, low-osmolar contrast media; IOCM, iso-osmolar contrast media.

We conducted a retrospective propensity score (PS)-matched study at a single center to analyze the difference between LOCM and IOCM in the development of CI-AKI. All patients who underwent coronary angiography (CAG) or percutaneous coronary intervention (PCI) between 1 January 2007, and 31 December 2016, were screened for eligibility for the study. The total number of screened patients was 12,742 and 7,475 patients had been excluded based on the following exclusion criteria: (1) patients without pre- and post-procedural laboratory findings (*n* = 6,141), (2) patients exposed to CM within 7 days before or 3 days after the procedure (*n* = 963), (3) patients with receiving dialysis before study entry (*n* = 301), (4) patients without data for the type of CM (*n* = 70). Therefore, 5,267 patients were included in the final analysis (Figure 1).

This study was approved by the Institutional Review Board of Gachon University Gil Medical Center (GCIRB2019-248). Because this study did not involve any further intervention in the retrospective analysis, the need for obtaining consent was waived by the IRB of Gachon University Gil Medical Center. All methods were performed as per relevant guidelines and regulations.

### Covariates

The patients' demographic, clinical, and laboratory data were obtained from electronic medical records. Demographic data included age, sex, body mass index (BMI), smoking status, comorbidities [diabetes, hypertension, and history of congestive heart failure (CHF)], and preprocedural prescript medication [renin-angiotensin-aldosterone system (RAAS) blockers [angiotensin-converting enzyme inhibitors (ACEi) or angiotensin receptor blockers (ARB)], beta-blockers, calcium channel blockers (CCB), diuretics, and statins]. We also collected the patients' clinical information about left ventricular ejection fraction (LVEF), left ventricular mass index (LVMI), blood pressure (BP) at the time of CAG, presence of multivessel disease, and the type and amount of contrast media used. Hypertension was defined as a documented blood pressure ≥ 140/90 mmHg or using anti-hypertensive medications. History of CHF or LVEF ≤40% on echocardiography was considered as heart failure.

Laboratory data included serum creatinine levels before and after CAG or PCI, as well as hemoglobin, albumin, cholesterol, triglyceride, high-density lipoprotein (HDL) cholesterol, and low-density lipoprotein (LDL) cholesterol levels at hospital admission. We calculated the estimated glomerular filtration rate (eGFR) using the Chronic Kidney Disease Epidemiology Collaboration (CKD-EPI) equation. Chronic kidney disease (CKD) was defined as either eGFR ≤60 ml/min/1.73 m^2^, proteinuria (defined as trace or greater by dipstick), or both on at least 2 occasions ≥ 3 months apart.

The Mehran CI-AKI risk scores were calculated for each patient from the corresponding scores for the 8 prognostic variables suggested in the previous report (10).

### Coronary Intervention and Contrast Media

Each patient followed the principle of hydration with isotonic saline for 12 h before and after exposure to CM, and the amount and rate were adjusted by the clinician according to the patient's tolerance. Each patient underwent CAG from the arterial access of either the femoral or radial arteries. The number of diseased coronary arteries was categorized as per the American Heart Association classifications. For significant stenotic coronary lesions, PCI was performed with a balloon catheter or stent.

The non-ionic monomeric LOCM, namely iopromide (774 mOsm/kg) or iopamidol (800 mOsm/kg), was used in 54.2% of the total patients. The others have used the non-ionic dimeric IOCM, iodixanol (290 mOsm/kg) for coronary catheterization.

### Primary Endpoint

The primary endpoint was CI-AKI defined as Kidney Disease Improving Global Outcome (KDIGO) criteria, an absolute increase of serum creatinine level ≥ 0.3 mg/dl or relative increase ≥ 50% from baseline creatinine level within 48 h after exposure to the CM.

### Statistical Analysis

Because the study was a retrospective observational study, we used the PS matching analysis to control any potential confounding and selection bias. A multivariable logistic regression analysis model was generated to predict the probability that LOCM would be administrated for the given covariates: age; sex; BMI; diabetes; hypertension; smoking status; CHF; baseline levels of eGFR, hemoglobin, albumin, cholesterol, triglycerides, HDL cholesterol, and LDL cholesterol; use of RAAS blockers, beta-blockers, CCBs, diuretics and statins; LVEF; LVMI; blood pressure; amount of CM used; presence of multivessel disease; PCI; and Mehran score. According to these covariates, a PS was calculated for each patient. And we used the derived PS values to match 2,263 IOCM users with LOCM users at a ratio of 1:1 using the nearest neighbor with calipers method (caliper = 0.1). After all PS matches were performed, we conducted the balance test in baseline covariates using the standardized mean difference, paired *t*-test, and McNemar's tests for continuous variables and categorical variables, respectively.

All continuous and categorical variables were expressed as the mean ± standard deviation and absolute counts with percentages, respectively. Before PS matching, continuous variables were compared by *t*-test and categorical variables were compared by the χ2 test. To determine independent risk factors for CI-AKI, a multivariable logistic regression analysis was used in the unmatched cohort and matched cohort, respectively. A logistic regression model stratified on clinically important variables, including a high Mehran score (≥6), was also used in the matched cohort. All statistical analyses were performed using R software, version 4.1.0 with packages (The Comprehensive R Archive Network: http://cran.r-project.org). Statistical significance was defined as *p* < 0.05.

## Results

The baseline characteristics of the study population are summarized in Table 1. The patients who were used LOCM were more likely to be male than the patients who were used IOCM. These patients were more likely to have diabetes, previous CHF, multivessel disease, low hemoglobin, high albumin, and were more likely to have prescribed peri-procedural medications (RAAS blockers, beta-blockers, CCBs, and diuretics). The BMI and cholesterol, triglyceride, and HDL- and LDL-cholesterol levels did not vary significantly between the two groups.

**Table 1:** Baseline characteristics of the study participants.

**Variable**	**Before matching**				**After propensity matching**			
	**LOCM users,**	**IOCM users,**		**Standardized**	**LOCM users,**	**IOCM users,**		**Standardized**
	**(*N* = 2,855)**	**(*N* = 2,412)**	**P**	**differences**	**(*N* = 2,263)**	**(*N* = 2,263)**	**P**	**differences**
Age, year	61.7 ± 12.8	61.5 ± 12.7	0.668	0.012	61.6 ± 12.7	61.5 ± 12.8	0.715	0.011
Male gender, n (%)	1,653 (57.9)	1,328 (55.1)	**0.041**	0.057	1,278 (56.5)	1,261 (55.7)	0.627	0.015
Diabetes, n (%)	332 (11.6)	225 (9.3)	**0.008**	0.075	221 (9.8)	218 (9.6)	0.919	0.004
Hypertension, n (%)	640 (22.4)	520 (21.6)	0.474	0.021	487 (21.5)	493 (21.8)	0.856	0.006
Smoking, n (%)	785 (27.5)	612 (25.4)	0.088	0.048	578 (25.5)	563 (24.9)	0.390	0.015
BMI, kg/m^2^	24.5 ± 2.9	24.6 ± 2.4	0.678	0.011	24.5 ± 2.9	24.6 ± 2.5	0.740	0.010
Previous CHF, n (%)	376 (13.2)	252 (10.4)	**0.003**	0.084	267 (11.8)	250 (11.0)	0.460	0.024
Baseline eGFR, ml/min/1.73 m^2^	73.4 ± 58.1	74.7± 39.6	0.320	0.027	74.5 ± 42.0	74.5 ± 39.7	0.978	0.001
CKD, n (%)	1,250 (43.8)	1,019 (42.2)	0.274	0.031	950 (42.0)	955 (42.2)	0.903	0.004
**Angiographic**
LV EF, %	57.1 ± 9.3	57.6 ± 8.9	**0.028**	0.061	57.3 ± 8.9	57.5 ± 8.9	0.490	0.020
LVMI, g/m^2^	105.2 ± 20.6	104.7 ± 19.8	0.309	0.028	105.1 ± 19.5	104.8 ± 19.9	0.559	0.017
SBP, mmHg	121.1 ± 16.6	120.8 ± 13.1	0.469	0.020	120.9 ± 15.6	120.9 ± 13.2	0.952	0.002
DBP, mmHg	74.2 ± 9.9	73.9 ± 7.8	0.202	0.035	74.0 ± 9.3	73.9 ± 7.9	0.714	0.011
Contrast volume, ml	155.4 ± 89.3	151.0 ± 84.2	0.068	0.050	150.2 ± 85.6	150.0 ± 83.5	0.894	0.002
**Coronary arterial disease**
Multivessel, n (%)	673 (23.6)	543 (22.5)	**<0.001**	0.092	491 (21.7)	495 (21.9)	0.817	0.004
No lesion	2,069 (72.5)	1,869 (77.5)			1,697 (75.0)	1,688 (74.6)		
1-vessel disease	113 (4.0)	80 (3.3)			75 (3.3)	80 (3.5)		
2-vessel disease	590 (20.7)	517 (21.4)			444 (19.6)	469 (20.7)		
3-vessel disease	83 (2.9)	26 (1.1)			47 (2.1)	26 (1.1)		
PCI, n (%)	571 (20.0)	485 (20.1)	0.950	0.003	425 (18.8)	437 (19.3)	0.677	0.014
Hemoglobin, g/dl	12.6 ± 2.0	12.8 ± 2.0	**<0.001**	0.098	12.7 ± 2.0	12.8 ± 2.1	0.397	0.037
Albumin, g/dl	3.9 ± 0.5	3.8 ± 0.5	**0.003**	0.082	3.9 ± 0.5	3.9 ± 0.5	0.217	0.001
Cholesterol, mg/dl	174.1 ± 40.6	173.8 ± 36.4	0.722	0.010	174.1 ± 39.5	174.0 ± 37.1	0.962	0.001
Triglycerides, mg/dl	151.4 ± 101.4	150.5 ± 90.5	0.747	0.009	151.6 ± 97.6	150.8 ± 92.5	0.757	0.009
HDL-cholesterol, mg/dl	44.8 ± 11.6	44.9 ± 11.4	0.675	0.012	45.0 ± 11.3	45.0 ± 11.7	0.915	0.003
LDL-cholesterol, mg/dl	99.0 ± 34.3	98.7 ± 32.9	0.712	0.010	98.8 ± 33.0	98.9 ± 33.6	0.932	0.003
**Preprocedural medications**
RAAS blockers, n (%)	1,877 (65.7)	1,483 (61.5)	**0.001**	0.089	1,440 (63.6)	1,428 (63.1)	0.732	0.011
Beta-blockers, n (%)	1,603 (56.1)	1,124 (46.6)	**<0.001**	0.192	1,156 (51.1)	1,117 (49.4)	0.240	0.034
CCB, n (%)	1,365 (47.8)	1,078 (44.7)	**0.026**	0.063	1,049 (46.4)	1,029 (45.5)	0.563	0.018
Diuretics, n (%)	1,084 (38.0)	822 (34.1)	**0.004**	0.081	803 (35.5)	793 (35.0)	0.776	0.009
Statin, n (%)	1,068 (37.4)	842 (34.9)	0.064	0.052	801 (35.4)	806 (35.6)	0.901	0.005
Mehran score	4.5 ± 3.8	3.9 ± 3.4	**<0.001**	0.149	4.1 ± 3.5	4.0 ± 3.5	0.434	0.023

*LOCM, low-osmolar contrast media; IOCM, iso-osmolar contrast media; BMI, body mass index; CHF, congestive heart failure; eGFR, estimated glomerular filtration rate; CKD, chronic kidney disease; LV EF, left ventricular ejection fraction; LVMI, left ventricular mass index; SBP, systolic blood pressure; DBP, diastolic blood pressure; PCI, percutaneous coronary intervention; HDL, high-density lipoprotein; LDL, low-density lipoprotein; RAAS blocker, renin-angiotensin-aldosterone system blocker; CCB, calcium channel blocker. The Bold values means P value under 0.05*.

**Figure 2:**
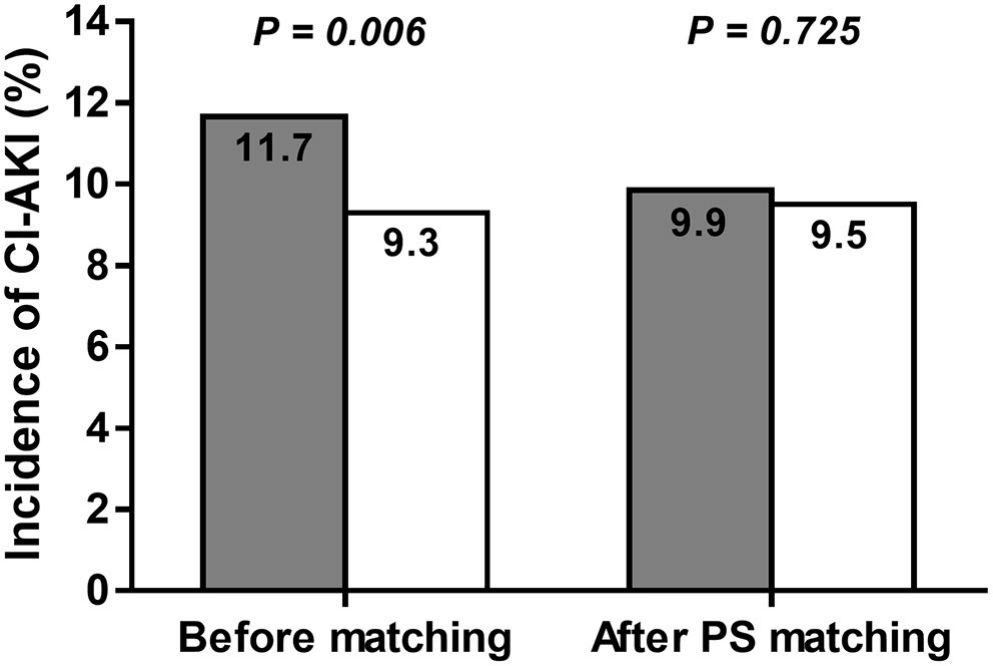
Incidence of contrast-induced acute kidney injury (CI-AKI) before and after propensity score (PS) matching in low-osmolar contrast media (LOCM) users (gray column) and iso-osmolar contrast media (IOCM) users (white column). The incidence of CI-AKI was not significantly different between the LOCM users and IOCM users after PS matching (9.9% vs. 9.5%; *p* = 0.725).

A total of 2,263 patients of the LOCM group were successfully matched to patients of the IOCM group (Figure 1). After PS matching, there were no statistically significant clinical differences between the LOCM users and IOCM users (Table 1). The LOCM users showed an increased incidence of CI-AKI (11.7% vs. 9.3%; *p* = 0.006), but it lost statistical significance after PS matching (9.9% vs. 9.5%, *p* = 0.725; Figure 2).

We performed logistic regression analyses to evaluate the type of CM as a risk factor for developing CI-AKI. The crude analysis showed that the increased risk of CI-AKI was associated with LOCM (odds ratio [OR] 1.290, 95% confidence interval [CI] = 1.079–1.542, *p* = 0.005; Table 2). In multivariable analyses, the adjusted ORs for CI-AKI in the LOCM group were 1.059 [95% CI = 0.875–1.282; *p* = 0.555] in unmatched cohort and 0.987 (95% CI = 0.803–1.214; *p* = 0.901) in matched cohort (Table 2).

**Table 2:** LOCM for CI-AKI on multivariable logistic regression analysis in the unmatched and matched cohorts.

**Adjusted models**	**Unmatched odds ratio (95% CI)**	***P***	**Matched odds ratio (95% CI)**	***P***
Crude	1.290 (1.079–1.542)	**0.005**	1.041 (0.855–1.268)	0.688
Model 1	1.279 (1.069–1.531)	**0.007**	1.038 (0.851–1.265)	0.714
Model 2	1.234 (1.029–1.480)	**0.023**	1.035 (0.848–1.260)	0.736
Model 3	1.160 (0.963–1.397)	0.118	1.017 (0.831–1.246)	0.868
Model 4	1.059 (0.875–1.282)	0.555	0.987 (0.803–1.214)	0.901

*Model 1: adjusted for demographics (age and gender)*.

*Model 2: adjusted for demographics and comorbidities (model 1 + smoking status, DM, hypertension, CKD and CHF)*.

*Model 3: adjusted for demographics, comorbidities, and medications (model 2 + RAAS blockers, CCBs, β-blockers, diuretics and statins)*.

*Model 4: adjusted for demographics, comorbidities, medications, and laboratory findings (model 3 + hemoglobin, albumin and contrast volume)*.

*DM, diabetes mellitus; CKD, chronic kidney disease; CHF, congestive heart failure; RAAS blocker, Renin-angiotensin-aldosterone system blocker; CCB, calcium channel blocker. The Bold values means P value under 0.05*.

**Figure 3:**
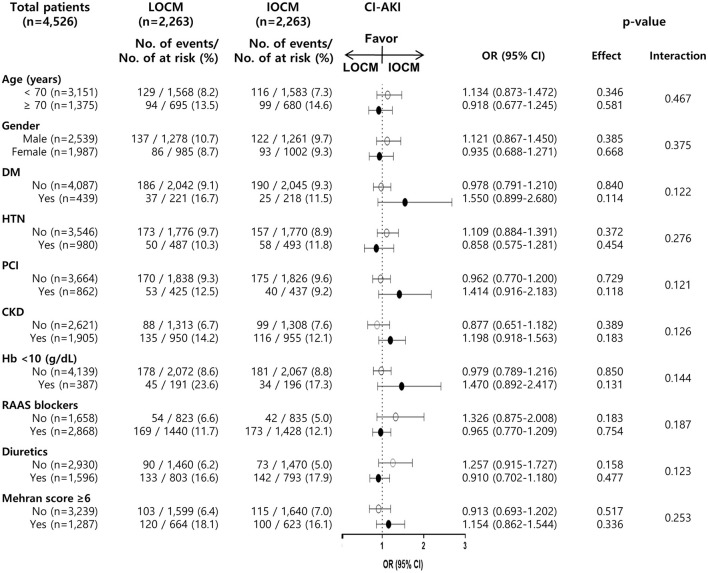
Association of LOCM use and development of CI-AKI in subgroups of the matched cohort. CI-AKI, contrast-induced acute kidney injury; LOCM, low-osmolar contrast media; IOCM, iso-osmolar contrast media; DM, diabetes mellitus; HTN, hypertension; PCI, percutaneous coronary intervention; CKD, chronic kidney disease; Hb, hemoglobin; RAAS blocker, renin-angiotensin-aldosterone system blocker.

We also performed a stratified analysis of clinically important variables. Of the 4,526 patients in the matched cohort, 1,905 (42.1%) were diagnosed with CKD. A total of 950 of 1,905 (49.9%) PS-matched CKD patients received LOCM, while 1,313 of 2,621 (50.1%) PS-matched non-CKD patients received LOCM (Figure 3). The ORs and 95% CI for CI-AKI among those with and without CKD were 1.198 (0.918–1.563, *p* = 0.183) and 0.877 (0.651–1.182, *p* = 0.389), respectively (*p* for interaction, 0.126, Figure 3). The associations between LOCM use and CI-AKI development were generally homogenous in the subgroups stratified by other variables (Figure 3).

As sensitivity analyses, we also analyzed the high-risk group for CI-AKI (Mehran score ≥6), but there was no difference in the incidence of CI-AKI between the LOCM and the IOCM groups (Figure 3).

## Discussion

In this study, we assessed the role of the types of CM in the development of CI-AKI among patients who underwent CAG or PCI. Using PS matching, we found that no significant difference existed in the occurrence of CI-AKI between LOCM and IOCM (9.9% vs. 9.5% respectively, *p* = 0.725). Furthermore, according to subgroup analyses, the types of administrated CM did not increase the occurrence of CI-AKI among patients who had risk factors for CI-AKI. These findings are expected to help provide useful information for selecting the types of CM in patients with CAG.

CM that was initially used had high ionicity and osmolality. It caused many non-renal complications, such as nausea, vomiting, and hypotension (11). In addition, it was considered that the high osmolality and intrinsic chemotoxicity of conventional ionic CM may be related to their nephrotoxic effects (12). For these reasons, researchers focused on the ionicity and osmolality of CM as key toxic properties and have tried to develop the optimal CM to reduce their chemotoxicity as well as improve diagnostic efficacy (13). After developing ionic and non-ionic LOCM in the 1980s, LOCM was widely used because osmo-toxicities of LOCM like nausea, vomiting, the pain of the injection site, and hemodynamic instability were improved compared to HOCM (13, 14). Furthermore, Barrett and Carlisle found that LOCM had less nephrotoxicity than HOCM in their meta-analysis (7). Because of the superiority of the LOCM, HOCM had deprecated and was replaced with LOCM (14). When IOCM was developed, according to the flow of such a concept of osmolality, many researchers tended to expect more excellent clinical outcomes in IOCM than LOCM in terms of nephrotoxicity.

However, it is controversial that IOCM has lower nephrotoxicity than LOCM. In the early randomized controlled trial known as the NEPHRIC study, Aspelin et al. (8) found that the IOCM group resulted in a smaller increase in serum creatinine and less likely to develop CI-AKI than the LOCM group in high-risk patients with CKD and diabetes. In the RECOVER study, Jo et al. (15) compared the nephrotoxicity of IOCM with ioxaglate, which is ionic dimeric LOCM among 275 CKD patients (creatinine clearance ≤60 ml/min) who underwent CAG with or without PCI. In that study, IOCM was associated with a lower incidence of CI-AKI compared with LOCM. However, in the CARE study (16), the rate of CI-AKI was not significantly different between the administration of IOCM and LOCM in 414 patients with eGFR of 20–59 ml/min.

Our observations are consistent with the findings of the CARE study and different from those of the NEPHRIC study and RECOVER study. This discrepancy needs to consider the other LOCM used in each study. In the NEPHRIC study, iohexol was assigned to the LOCM group, while ioxaglate was assigned to the LOCM group in the RECOVER study. In the CARE study, in common with our study, iopamidol was assigned to the LOCM group.

The different results from the type of LOCM can be found through some meta-analysis studies. A meta-analysis by McCullough et al. (17) demonstrated that IOCM had less nephrotoxicity compared with LOCM, especially in the patients with CKD and diabetes. In the pooled patient data, iohexol and ioxaglate were administrated to 87% of the 1,345 patients given LOCM, and iopamidol and iopromide were administrated to 13% of the LOCM group. Another meta-analysis by Heinrich et al. (18) found that the risk of CI-AKI did not reduce significantly when using IOCM compared with non-ionic LOCM except iohexol. In addition, a meta-analysis conducted by Zhang et al. (19) analyzed 8 randomized controlled trials and demonstrated that iodixanol was associated with a non-significantly lower risk of CI-AKI and significantly lower risk of a cardiovascular adverse event as compared with iopamidol. On the other hand, when 11 trials using iohexol were analyzed separately, IOCM (iodixanol) was related to lower nephrotoxicity than iohexol in that study. In addition, a study by Reed et al. (20) also confirmed those results. They showed that the use of iohexol and ioxaglate was more likely to increase the risk of CI-AKI than other LOCM as well as IOCM.

According to the result of those meta-analysis studies, it appears that there were differences between the types of LOCM. In particular, in the studies that assigned iohexol and ioxaglate to LOCM, clinical outcomes tend to be worse than those of IOCM, so additional studies on these CMs will help to obtain more information. Although a meta-analysis by Eng et al. (21) concluded that there were no differences in CI-AKI risk among types of LOCM, the number of studies and total sample size of the pooled patients were small and the strength of evidence was low. One possible explanation for the different nephrotoxicity among LOCM is its various chemical characteristics. Ioxaglate is the only ionic dimeric LOCM and iohexol has similar viscosity compared to IOCM (14, 22). Iopamidol and iopromide have a lower viscosity than IOCM (22). According to some animal studies, higher viscosity of CM was associated with an increased urine viscosity, increased GFR, and slower elimination of CM from the kidney (23, 24). Those animal studies indicated that iohexol could be associated with more nephrotoxicity than other LOCM and why other LOCM having lower viscosity than IOCM are not associated with a higher risk of CI-AKI. However, as for ioxaglate and iohexol, we were not able to determine whether these LOCM increase the risk of CI-AKI compared with IOCM or other LOCM because iopromide and iopamidol were used in the study. Therefore, we need to obtain more information through further study.

In previous studies, the risk factors of CI-AKI included pre-existing CKD, diabetes, high volume of CM, repeated administration of CM within 72 h, advanced age, anemia, use of RAAS blockers, dehydration, and patients' clinical presentation (4, 25–27). In the present study, multivariable logistic regression analysis in matched cohort showed that risk factors of CI-AKI are consistent with the previous studies (Supplementary Table 1).

CKD is one of the strongest risk factors for CI-AKI (28) and the incidence of CI-AKI increased from below 2% in patients with normal kidney function to 50% or more in patients with advanced kidney disease (29). For this reason, many previous studies were conducted on patients with CKD. In the present study, the rate of CI-AKI in patients with CKD was 13.6% in a matched cohort group. In subgroup analysis, our observations showed that there was no significant difference between the types of CM among patients with or without CKD (Figure 3).

The study had some limitations. First, this study was an observational study in a single-center, rather than a randomized controlled trial. Although using PS matching to minimize the difference in the basic characteristics between the two study groups, unmeasured covariates, such as clinical presentation, including symptomatic or stable angina, unstable angina, and non-ST-elevation myocardial infarction (NSTEMI) or STEMI, may have an influence on the results. Second, patients in the study were included regardless of their risk factors in contrast that most randomized controlled trials included high-risk patients. Considering that the incidence of CI-AKI in patients with normal kidney function was under 2%, the effect of the types of CM might be likely to be underestimated. Third, pre-existing AKI may be included at the start of the observation because the occurrence of AKI was estimated based on serum creatinine at the time of admission for CAG. Despite these limitations, our study is the only large observational cohort study using PS matching that provides evidence that no significant difference existed in the occurrence of CI-AKI between LOCM and IOCM.

## Conclusion

In conclusion, although the role of CM types in the development of CI-AKI has been debated, our observation shows that the selection between LOCM and IOCM during CAG has no influence on the incidence of CI-AKI. These findings are expected to help provide useful information for selecting the types of CM in patients with CAG.

## Data Availability Statement

The raw data supporting the conclusions of this article will be made available by the authors, without undue reservation.

## Ethics Statement

The studies involving human participants were reviewed and approved by the Institutional Review Board of Gachon University Gil Medical Center. Written informed consent for participation was not required for this study in accordance with the national legislation and the institutional requirements. Written informed consent was not obtained from the individual(s) for the publication of any potentially identifiable images or data included in this article.

## Author Contributions

JYJ conceived and designed the experiments. TL and JYJ contributed to data analysis/interpretation and manuscript writing. Each author provided important intellectual content by presenting and solving questions about the accuracy or integrity of all parts of the work. All authors contributed to data acquisition, reviewing of the draft, and approved the final version of the manuscript.

## Conflict of Interest

The authors declare that the research was conducted in the absence of any commercial or financial relationships that could be construed as a potential conflict of interest.

## Publisher's Note

All claims expressed in this article are solely those of the authors and do not necessarily represent those of their affiliated organizations, or those of the publisher, the editors and the reviewers. Any product that may be evaluated in this article, or claim that may be made by its manufacturer, is not guaranteed or endorsed by the publisher.
