# Supplementary material for: Low-Osmolar vs. Iso-Osmolar Contrast Media on the Risk of Contrast-Induced Acute Kidney Injury: A Propensity Score Matched Study
Source: Front Med (Lausanne). 2022 Apr 29;9:862023. doi: 10.3389/fmed.2022.862023 (PMC9099141; doi:10.3389/fmed.2022.862023)
Supplement: Supplementary file 1 [file Table_1.pdf]

Supplementary Table 1. Univariate and multivariate associations of individual variables with CI-AKI in the matched cohort

Variable	Univariate odds ratio (95% CI)	P	Multivariate odds ratio (95% CI)	P
LOCM	1.041 (0.855 – 1.268)	0.688		
Diuretics	3.534 (2.880 – 4.336)	**<0.001**	2.820 (2.241 – 3.556)	**<0.001**
Hemoglobin < 10 g/dl	2.701 (2.062 – 3.537)	**<0.001**	1.830 (1.351 – 2.478)	**<0.001**
Albumin < 3.5 g/dl	2.407 (1.930 – 3.002)	**<0.001**	1.810 (1.410 – 2.313)	**<0.001**
CKD	1.975 (1.618 – 2.411)	**<0.001**	1.520 (1.231 – 1.879)	**<0.001**
RAAS blockers	2.203 (1.740 – 2.787)	**<0.001**	1.340 (1.031 – 1.753)	**0.029**
Contrast volume, 100 ml increase	1.034 (1.023 – 1.045)	**<0.001**	1.040 (1.029 – 1.053)	**<0.001**
LV EF < 40%	1.729 (1.177 – 2.539)	**0.005**	1.330 (0.881 – 2.009)	0.174
DM	1.623 (1.216 – 2.167)	**0.001**	1.290 (0.949 – 1.745)	0.105
Beta-blockers	1.537 (1.258 – 1.879)	**<0.001**	1.090 (0.874 – 1.354)	0.452
Age, yr	1.028 (1.020 – 1.037)	**<0.001**	1.010 (1.000 – 1.018)	0.057
HTN	1.207 (0.959 – 1.519)	0.109		
Male gender	1.147 (0.939 – 1.402)	0.178		
Smoking	1.134 (0.908 – 1.415)	0.268		
Statin	0.994 (0.809 – 1.222)	0.957		
CCB	0.857 (0.702 – 1.046)	0.128		

LOCM, low-osmolar contrast media; CKD, chronic kidney disease; RAAS, renin-angiotensin-aldosterone system; LV EF, left ventricular ejection fraction; HTN, hypertension; CCB, calcium channel blocker.
